# Closed-Loop Recyclable and Nonpersistent Polyethylene-like Polyesters

**DOI:** 10.1021/acs.accounts.3c00811

**Published:** 2024-03-06

**Authors:** Marcel Eck, Stefan Mecking

**Affiliations:** Chair of Chemical Materials Science, Department of Chemistry, University of Konstanz, Universitätsstrasse 10, 78457 Konstanz, Germany

## Abstract

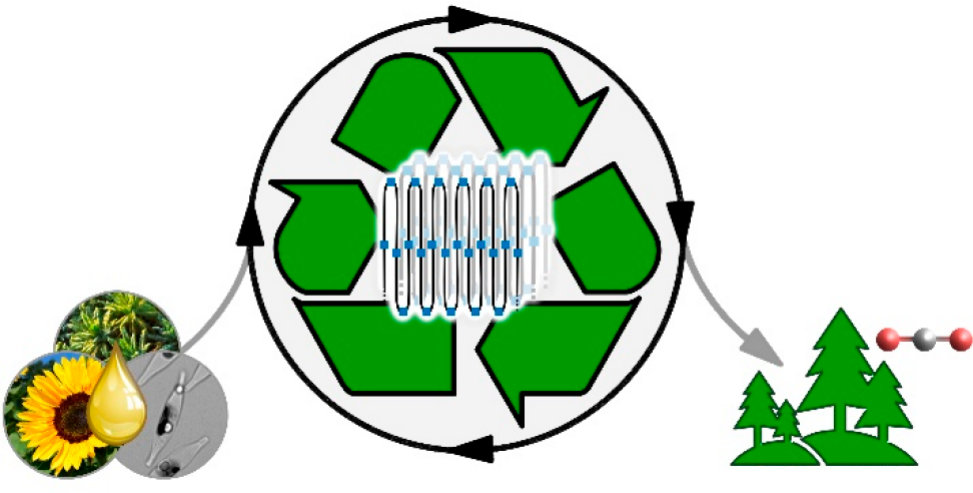

Aliphatic polyesters based on long-chain monomers were synthesized for the first time almost a century ago. In fact, Carothers’ seminal observations that founded the entire field of synthetic polymer fibers were made on such a polyester sample. However, as materials, they have evolved only over the past decade. This is driven by the corresponding monomers becoming practically available from advanced catalytic conversions of plant oils, and future prospects comprise a possible generation from third-generation feedstocks, such as microalgae or waste. Long-chain polyesters such as polyester-18.18 can be considered to be polyethylene chains with a low density of potential breakpoints in the chain. These do not compromise the crystalline structure or the material properties, which resemble linear high-density polyethylene (HDPE), and the materials can also be melt processed by injection molding, film or fiber extrusion, and filament deposition in additive manufacturing. At the same time, they enable closed-loop chemical recycling via solvolysis, which is also possible in mixed waste streams containing polyolefins and even poly(ethylene terephthalate). Recovered monomers possess a quality that enables the generation of recycled polyesters with properties on par with those of the virgin material. The (bio)degradability varies enormously with the constituent monomers. Polyesters based on short-chain diols and long-chain dicarboxylates fully mineralize under industrial composting conditions, despite their HDPE-like crystallinity and hydrophobicity. Fundamental studies of the morphology and thermal behavior of these polymers revealed the location of the in-chain groups and their peculiar role in structure formation during crystallization as well as during melting. All of the concepts outlined were extended to, and elaborated on further, by analogous long-chain aliphatic polymers with other in-chain groups such as carbonates and acetals. The title materials are a potential solution for much needed circular closed-loop recyclable plastics that also as a backstop if lost to the environment will not be persistent for many decades.

## Key References

- NelsonT. F.; RothauerD.; SanderM.; MeckingS.Degradable and Recyclable Polyesters from Multiple Chain Length Bio- and Waste-Sourceable Monomers. Angew. Chem., Int. Ed.2023, 62, e20231072910.1002/anie.20231072937675615.^1^*Polyesters from waste or biomass sourceable multiple-chain-length dicarboxylic acids adopt HDPE-like solid-state structures, alike single-chain-length congeners. The materials can be injection molded or extruded to films and fibers.*^*13*^*C-labeling biodegradation studies show rapid mineralization in soil*.
- EckM.; SchwabS. T.; NelsonT. F.; WurstK.; IberlS.; SchleheckD.; LinkC.; BattagliarinG.; MeckingS.Biodegradable High-Density Polyethylene-like Material. Angew. Chem., Int. Ed.2023, 62, e20221343810.1002/anie.202213438PMC1010771236480133.^2^*HDPE-like polyester material PE-2,18, generated from readily available biobased long-chain C*_*18*_*-diacid and short-chain ethylene glycol, despite its high crystallinity is biodegraded in industrial composting within two months. Studies of PE-18,18 indicate a strong influence of the diol component on biodegradability*.
- HäußlerM.; EckM.; RothauerD.; MeckingS.Closed-loop recycling of polyethylene-like materials. Nature2021, 590, 423–42710.1038/s41586-020-03149-933597754 .^3^*Polyethylene-like polycondensates can be recycled in a closed loop via depolymerization. Alcoholysis under mild conditions (120 °C) quantitatively yields pure recovered monomers. Repolymerized polymers’ properties are on par with virgin materials, as exemplefied through biobased polyester PE-18,18 and polycarbonate PC-18*.
- WittT.; HäußlerM.; KulpaS.; MeckingS.Chain Multiplication of Fatty Acids to Precise Telechelic Polyethylene. Angew. Chem., Int. Ed.2017, 56, 7589–759410.1002/anie.20170279628472549.^4^*Ultra-long-chain α,ω-difunctional building blocks are obtained by a sequence of two scalable catalytic steps that virtually double the chain length of the fatty acid feedstock. Resulting PE-48,48s’ unprecedented melting point (T*_*m*_*= 120 °C) surpasses that of low-density polyethylene (LDPE)*.

## Introduction

Aliphatic polyesters with long methylene repeat units contributed significantly to the seminal developments that founded today’s polymer technology. Carothers’ groundbreaking paper on synthetic fibers in which he outlined the principles of cold drawing was centered on polyester-3,16 (PE-3,16) as the object of study, that is, a polyester based on linear hexadecanedioic acid (C_16_-dicarboxylic acid) and 1,3-propanediol (C_3_-diol).^5^ In the following decades, long-chain polycondensates have received comparatively little attention, likely due to the limited availability of the monomers. Studies were largely limited to the elucidation of the dependence of melting points on the repeat unit chain length of aliphatic polyesters. The emergence of long-chain polyesters as materials in this century was driven by the need for renewable alternatives to petroleum-based materials and the prospect of biodegradability, although this was elaborated on only recently. Recyclability as a strong driver has come up in the past few years.

Related polyhydroxyalkanoates (PHA) have been developed as biodegradable and renewable plastics since the 1960s. They are harvested from bacteria, in which they serve as energy storage, and can additionally be sourced from genetically engineered plants.^6a−6c^ Although longer-chain versions can be generated, material developments have focused on short-chain poly(hydroxy butyrate) (PHB) and poly(hydroxy butyrate-*co*-valerate) (PHBV).^7^ Ring-opening polymerization of pentadecalactone catalyzed by enzymes or by synthetic small-molecule catalysts yields a linear long-chain AB-type polyester,^8^ which was studied in depth as a polyethylene-like material with properties intermediate to low-density polyethylene (LDPE) and high-density polyethylene (HDPE) especially by Duchateau et al.^9−11^ Pentadecalactone occurs naturally but is usually produced in five steps from petrochemical feedstocks.^12^ Long-chain ω-hydroxy fatty acids, suitable as AB-monomers for polyesterification, were generated by Gross et al. with engineered yeast strains.^13,14^

The advent of commercial sources of dicarboxylic acids in the past few years has accelerated the development of the field. Wilmar produces octadecanedicarboxylic acid (C_18_-diacid) via olefin metathesis of palm oil feedstock in a biorefinery in Indonesia, a technology that could also be applied to other feedstocks such as soy oil.^15^ Shanghai-based Cathay Biomaterials produces long-chain C_14_-, C_16_-, and C_18_-diacids by fermentation^16^ methods. Capacities for these different diacids are not disclosed, but combined current production volumes are likely on the multihundred ton scale or higher.

**Figure 1:**
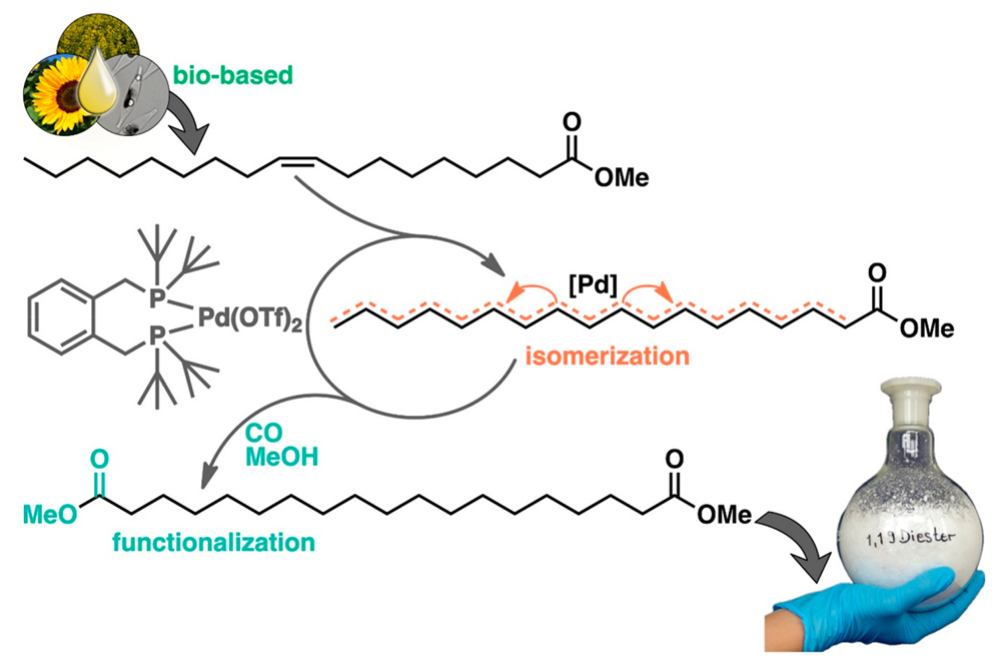
Schematic overview of the [Pd(dtbpx)(OTf)_2_]-catalyzed isomerizing methoxycarbonylation of traditional plant oils, waste, or algae oils’ unsaturated fatty acid esters to long-chain aliphatic diester monomers, illustrated here for methyl oleate feedstock (OTf = triflate). Adapted with permission from ref (23). Copyright 2014, Royal Society of Chemistry.

Our entry into the field was initiated by studies of carbonylation chemistry which provided novel long-chain monomers.^17^ Presentation of this chemistry and the resulting polyesters at the third Workshop on Fats and Oils as Renewable Feedstock for the Chemical Industry, organized by Mike Meier and Jürgen Metzger, in March 2010 met with an encouraging and motivating response. A Pd(II) catalyst coordinated by the bulky diphosphine 1,2-*bis*(di-*tert*-butylphosphino)xylene (dtbpx) is employed in the industrial methoxycarbonylation of ethylene to methylpropionate, which serves as a precursor for methyl methacrylate. With internal olefins as a substrate, the new ester group is generated not at the original site of the double bond but in a terminal position. That is, with an oleate as a substrate, the ester group is generated eight carbon atoms away from the double bond’s initial position (Figure 1).^17,18^ Mechanistic studies revealed that this unusual isomerizing carbonylation is due to a rapid migration of the catalytically active site up and down the fatty acid substrates’ chain along with rapid carbon monoxide insertion and deinsertion events.^19,20^ The terminal selectivity (up to 95% at 95% conversion) originates from a lower barrier of methanolysis, which is the rate-determining step, at the less sterically demanding linear acyl. Also, technical-grade feedstocks such as high oleic sunflower oil, tall oil, or microalgae oil can be converted.^21^ The substantial amounts of multiple unsaturated fatty acids in the latter slow down the isomerizing carbonylation by the formation of stable allyl complexes.^20^ This can be overcome by selective hydrogenation in a tandem catalysis reaction as illustrated for microalgae oils from wild-type strains as well as from genetically engineered strains that produce a different chain length and degree of unsaturation distribution.^22^ Isomerizing methoxycarbonylation of high oleic sunflower oil or ethyl erucate provided a ready access to the corresponding linear diester (C_19_ or C_23_), respectively, on a multi-100-g scale in high purity as required for polycondensation reactions.^23^

## Material Properties

**Figure 2:**
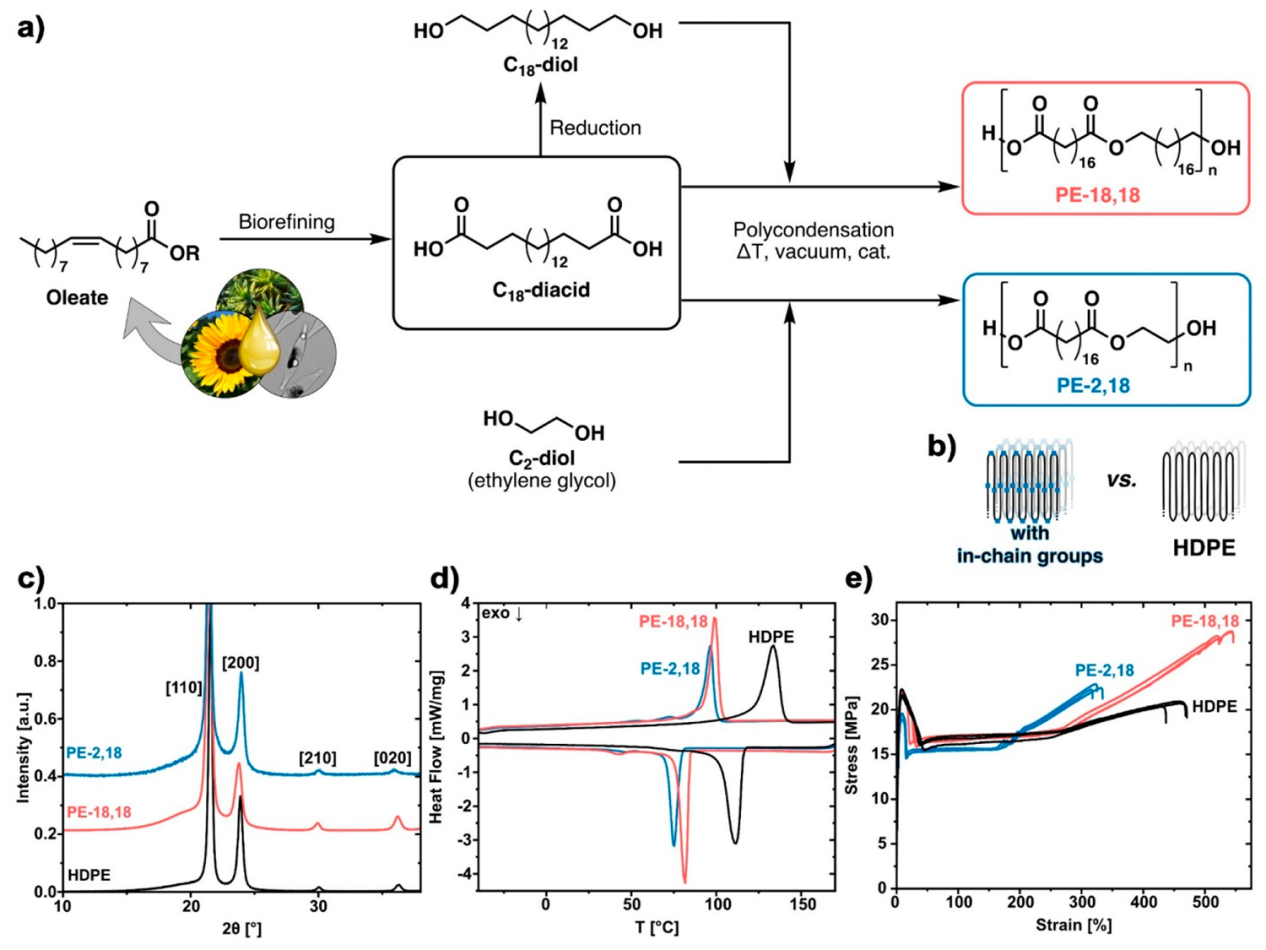
Synthesis and characterization of polyethylene-like polyesters. (a) Synthesis of polyethylene-like polyesters by A_2_ + B_2_ polycondensation starting from biorefining of plant or microalgae oils, exemplified for PE-18,18 and PE-2,18. (b) Schematic representation of the solid-state structure of polyethylene-like polymers with in-chain functional groups (left) and of HDPE (right). (c) WAXS diffractograms of PE-18,18 and PE-2,18 in comparison to HDPE. (d) DSC traces of PE-18,18 and PE-2,18 in comparison to HDPE. (e) Stress–strain curves of PE-18,18 and PE-2,18 in comparison to HDPE. Adapted with permission from ref (3). Copyright 2021, Springer Nature.

**Figure 3:**
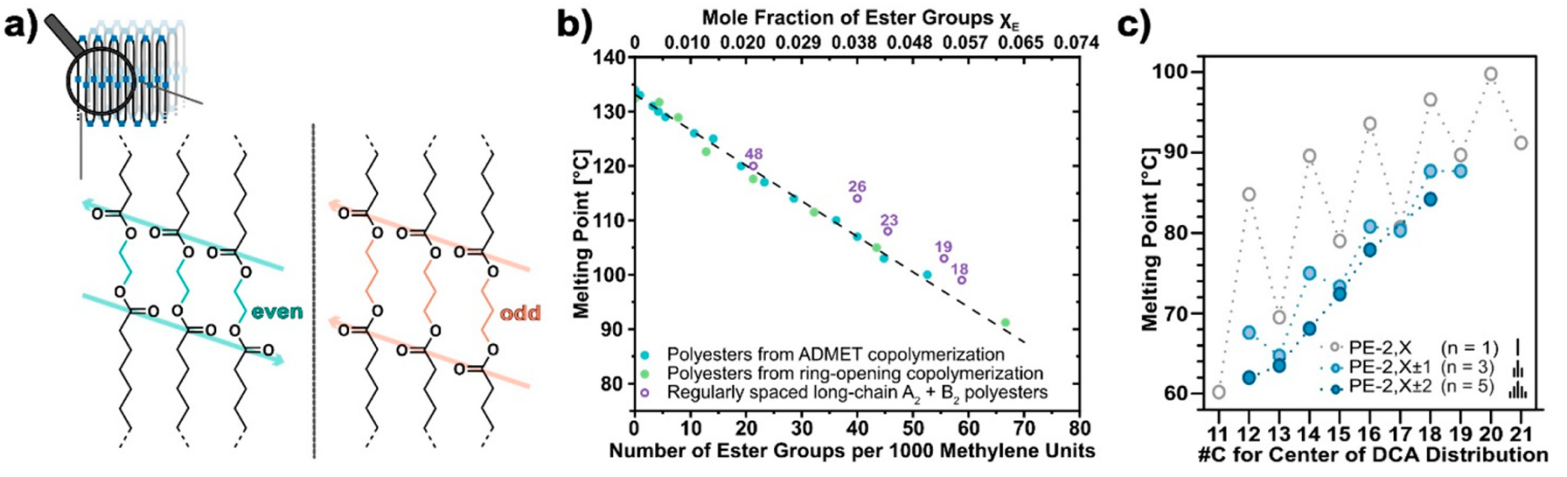
Melting points of long-chain polyesters. (a) Schematic illustration of the arrangement of the polar layers in aliphatic polyesters with an even number of carbons between the ester groups (left) and an odd number of carbons between ester groups (right) to account for the observed trends in the melting behavior. Exemplified for polyesters from a short-chain diol and long-chain dicarboxylic acid. The arrows indicate the directions of polarization. Adapted with permission from ref (3). Copyright 2021, Springer Nature. (b) Melting point vs density of ester groups in the chain. Data for polyesters with a largely random ester group distribution (blue symbols, polymers from ADMET/exhaustive hydrogenation; green symbols, polymers from ROMP/exhaustive hydrogenation; data from both synthesis methods agrees) and for regularly spaced polyesters-X,X (violet open symbols, number indicates the repeat unit carbon number X). (c) Peak *T*_m_ for polyesters-2,X, showing the pronounced odd–even effects (gray, open symbols) and for polyesters from mixtures of three dicarboxylic acids (i.e., center ±1; light blue) or five dicarboxylic acids (i.e., center ±2; dark blue). Adapted with permission from ref (1). Copyright 2023, Wiley-VCH Verlag GmbH *&* Co. KGaA.

Long-chain polycondensates were compared to polyethylenes (LDPE or HDPE) early on, and termed as “polyethylene-like”.^24,25^ The criteria for likeness to polyethylene are debatable; one straightforward and reasonable argument is the solid-state structure. Wide-angle X-ray scattering shows that long-chain polyesters commonly possess the same orthorhombic unit cell and crystal structure as found for linear polyethylene (Figure 2c). This is already the case for PE-2,11^26^ and essentially translates to the crystalline structure being dominated by the hydrocarbon chains’ order and arrangement, as in typical HDPE folded-chain crystallites. Ester groups are located in the amorphous portions but can also be accommodated in the crystal as demonstrated by Schmidt-Rohr et al. for PE-22,4 (Figure 2b).^27,28^ The latter goes along with an energy penalty,^29^ ε, which translates to lower melting points compared to HDPE (Figure 2d). This energy penalty can be partially compensated by a favorable, layered arrangement^30−32^ of the dipole moments of carbonyl groups of adjacent packed chains. The formation of these layered structures and the number of layers in the crystal can depend on the crystallization conditions, among other factors. This structure is also the origin of well-known odd–even effects,^33^ which are particularly pronounced for short distances between ester groups in the chain as given by combinations of short-chain and long-chain diols and dicarboxylates, respectively. In this case, the dipole moments of adjacent carbonyl groups in the chain can compensate for each other or not (Figure 3a). Notably, also long-chain polyesters from mixtures of dicarboxylate monomers of variable length adopt polyethylene-like structures as observed for polyesters PE-2,X ± Y (with 2 referring to the C_2_ length of the utilized diol ethylene glycol and X referring to the C_X_ average length of the utilized mixture of dicarboxylates which have multiple different chain lengths that vary from the average by a value of Y).^1^ Odd–even effects are absent in these polyesters as the irregular spacing of ester groups in the chain hinders favorable dipole alignment, and their melting points are similar to those of the “mismatched” single-length monomer polyesters PE-2,X from odd-numbered dicarboxylates (Figure 3c). Such polyesters from dicarboxylate mixtures are also of interest as the latter can be potentially sourced from low-value biomass or plastic waste.

The effect of in-chain ester groups can be modeled conveniently by polymers derived from acyclic diene metathesis (ADMET)^34^ or ring-opening metathesis (ROMP) copolymerization followed by exhaustive double-bond hydrogenation.^35,36^ ADMET copolymerization of an ester-centered α,ω-diene monomer such as undec-10-en-1-yl undec-10-enoate with an unfunctionalized α,ω-diene allows for facile modification of the density of in-chain functional groups in the regime of relatively low functional groups per methylene, which is of interest here (i.e., 53 down to 0 ester units per 1000 methylene units). The observed melting-point depression qualitatively agrees with the Sanchez–Eby inclusion model^29^ and decreases linearly vs linear polyethylene (PE) with an increasing mole fraction of ester units randomly distributed within the polymer chain (*T*_m_ = (133–683×*X*_E_) °C; *X*_E_ is the mole fraction of ester units; Figure 3b). Interestingly, regularly spaced polyesters, generated, for example, by A_2_ + B_2_ polycondensation, exhibit slightly higher melting points than their ADMET counterparts with a random distribution of ester groups. This can be ascribed to the greater ability of regularly spaced polyesters to form layers of ester groups by dipole–dipole interactions (cf. the odd–even effect), reducing the energy penalty ε caused by the incorporation of ester groups into the crystalline lamellae.

The aforementioned picture is further confirmed by ADMET model polymers with other in-chain functional groups. Akin to ester moieties, carbonate^37^ and keto^38,39^ groups are included in the PE-like orthorhombic crystalline phase. However, due to different dipole moments in comparison to ester groups (μ_carbonate_ = 0.91 D < μ_ester_ = 1.75 D < μ_ketone_ = 2.70 D), the ability to partially compensate for the resulting energy penalty by dipolar interactions varies. This results in a stronger melting-point depression for polycarbonates (*T*_m_ = (133–1033×*X*_C_) °C; *X*_C_ is the mole fraction of carbonate units) and a weaker melting-point depression for polyketones (*T*_m_ = (133–172×*X*_K_) °C; *X*_K_ is the mole fraction of keto units) compared to polyesters. In-chain acetal^37,40^ and amide^41^ groups exhibit a perturbing effect on PE crystals, and orthorhombic solid-state structures are obtained only for low functional group content. For the particularly interesting case of long-chain polyamides,^42,43^ the transition from a polyethylene-like to a hydrogen-bond-dominated structure occurs at a density of only 35 amide groups per 1000 methylene units.^41^

**Figure 4:**
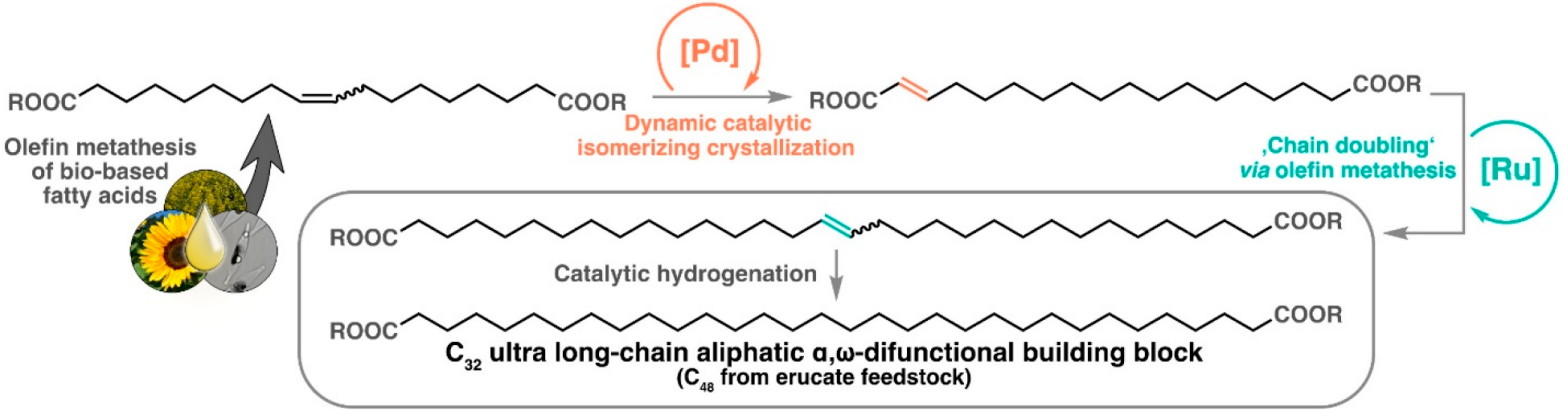
Schematic overview of chain doubling for the generation of ultra-long-chain aliphatic α,ω-difunctional building blocks, shown for the example of an oleate feedstock.

Other than ADMET, A_2_ + B_2_ polyesterification (Figure 2a) as a method is proven on an industrial scale, and C_12_–C_26_ building blocks are commercially available or can be produced by scalable catalytic methods, such as isomerizing alkoxycarbonylation (Figure 1) or the self-metathesis of fatty acids.^44,45^ Long-chain polyesters derived from these monomers contain as few as 40 (PE-26,26) ester groups per 1000 methylene units corresponding to a melting point of 114 °C for PE-26,26. To further approach the melting point of HDPE (*T*_m_ ≈ 130 °C), monomer building blocks that exceed the carbon number length of a typical fatty acid chain are desirable. These can be generated by a cycle of self-metathesis, isomerization, and again net self-metathesis (Figure 4). The key step is a dynamic catalytic isomerizing crystallization that selectively converts the internal unsaturated fatty acid self-metathesis product to the α,β-unsatured isomer.^4^ This entirely catalytic chain-doubling approach provides ultralong-chain monomers, namely, C_32_- and C_48_-diesters from oleate and erucic feedstock, respectively. Ti-catalyzed polycondensation of the C_48_-diester with the corresponding diol gave PE-48,48 which exhibits an unrivaled melting point of 120 °C, surpassing the melting transition of (branched) LDPE. The C_48_ molecules themselves possess a sufficient chain length to crystallize in polyethylene-like lamellae and can be considered to be telechelic polyethylenes with perfect end group fidelity.

**Figure 5:**
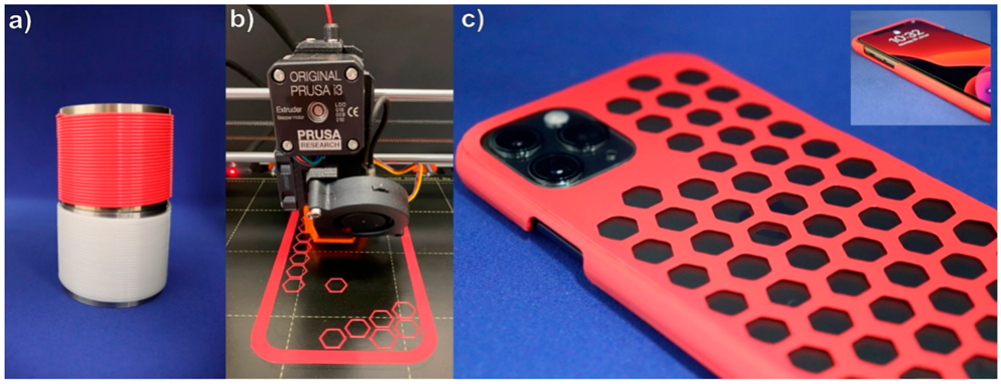
Filament fabrication and 3D printing of PE-18,18. (a) Red-colored and natural PE-18,18 filament. (b) Fused filament fabrication of a mobile phone protective cover using a red PE-18,18 filament. (c) Mobile phone protective cover 3D printed demonstration object from PE-18,18 enclosing a smartphone. Reprinted with permission from ref (3). Copyright 2021, Springer Nature.

In addition to the solid-state structure, mechanical properties of long-chain polyesters are a second compelling argument for PE-likeness, which is especially important for material applications (Figure 2e). PE-18,18, a prominent representative of this class of materials derived from commercially available C_18_-diacid (Figure 2a), is characterized by high stiffness and ductility, and its tensile properties compare to those of commercial HDPE (Representative values for PE-18,18^46^ are Young’s modulus *E* ≈ 900 MPa, stress at yield σ_*y*_ ≈ 22 MPa, and elongation at break ε ≈ 500%; comparative literature values for HDPE^47^ are *E* ≈ 900 MPa, σ_*y*_ ≈ 27 MPa, and ε ≈ 900%). The similarity of mechanical properties in comparison to HDPE is based on the similar orthorhombic solid-state structure and a comparable degree of crystallinity (χ ≈ 70%, determined via wide-angle X-ray scattering (WAXS)).^47^ Note that the entanglement molar masses, which need to be significantly exceeded for melt processing and mechanical strength, are somewhat higher for these long-chain polyesters vs HDPE (entanglement molar mass *M*_e,PE-18,18_ or *M*_e,PE-2,18_ ≈ 4–5 kg mol^–1^^48^ vs *M*_e,HDPE_ ≈ 1–2 kg mol^–1^^49^). HDPE-like polyesters are processable by techniques widespread in the plastics industry, including compounding, injection molding, fiber spinning, and film extrusion.^23^ Oxygen and water vapor barrier properties are relevant, particularly for packaging applications. To this end, studies on melt-pressed films of crystalline long-chain polyesters demonstrated that oxygen permeabilities can be comparable to polyolefins, while water vapor permeabilities are significantly improved in comparison to poly(butylene adipate-*co*-terephthalate) (PBAT), an aliphatic-aromatic copolyester employed in packaging films.^26^ Note that as a complementary processing method long-chain polycondensates can also be well suited for additive manufacturing (fused filament fabrication, FFF), as shown through the examples of PE-18,18 and PC-18 (Figure 5).^3^ The application profile of HDPE-like polyesters can further be expanded by blending with compatible polymers, including HDPE and fillers.^3,23,50^

**Figure 6:**
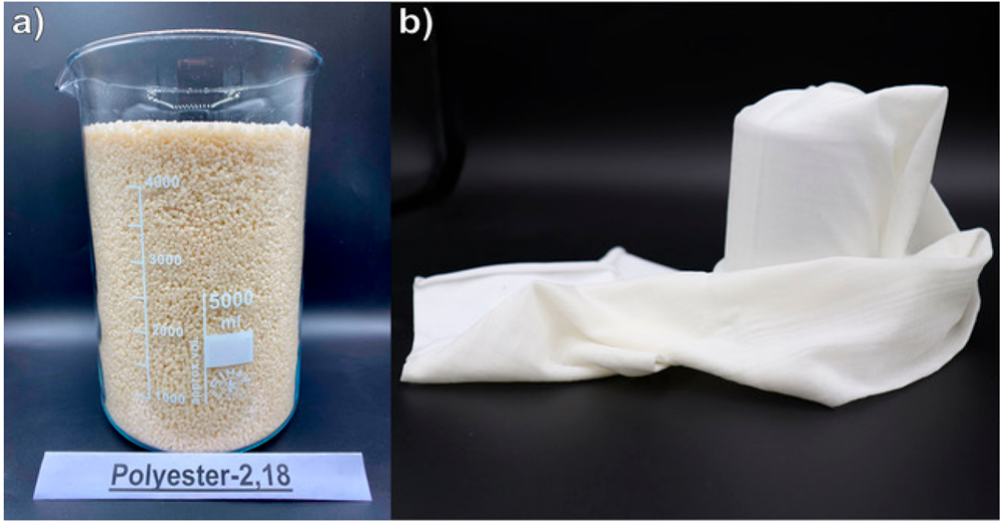
Upscaling and processing of PE-2,18. (a) PE-2,18 pellets obtained from a 10 kg-scale polycondensation reaction employing commercially available, technical grade monomers. (b) Fabric made from melt-spun PE-2,18 fibers.^51^

Compared to the long-chain diols (e.g., C_18_) employed in the aforementioned polyesters, short-chain diols can offer the advantage of commercial availability, and their water-solubility can also enhance the biodegradation process. Polyester-2,18 retains an HDPE-like solid-state structure (Figure 2c), and the material’s high crystallinity (χ ≈ 70%) is reflected in ductile tensile properties (*E* = 730 MPa, σ_*y*_ = 19 MPa, and ε = 330%) (Figure 2e).^2^ Its melting point (*T*_m_ = 96 °C vs *T*_m,PE-3,18_ = 82 °C and *T*_m,PE-4,18_ = 86 °C) is similar to that of its long-chain diol congener PE-18,18 (Figure 2d). Further studies therefore focused on this novel polyester material, which was accessible with high molar masses (up to *M*_n_ ≈ 120 kg mol^–1^), also facilitating processing by more demanding techniques such as melt spinning to yield synthetic fibers for textile applications (Figure 6).

## Recycling

Chemical recyclability via depolymerization to monomers is arguably a key element of a circular plastics economy in view of the limitations of mechanical recycling alone.^52,53^ Polyesters in general are attractive in this regard due to their amenability for solvolysis, as underlined by the industrial technology for PET recycling to terephthalic acid or ester monomers.^53^ By contrast, breaking down the inert hydrocarbon chains of polyethylene is unselective and requires high temperatures, with low yields of recovered ethylene monomer.^54^

**Figure 7:**
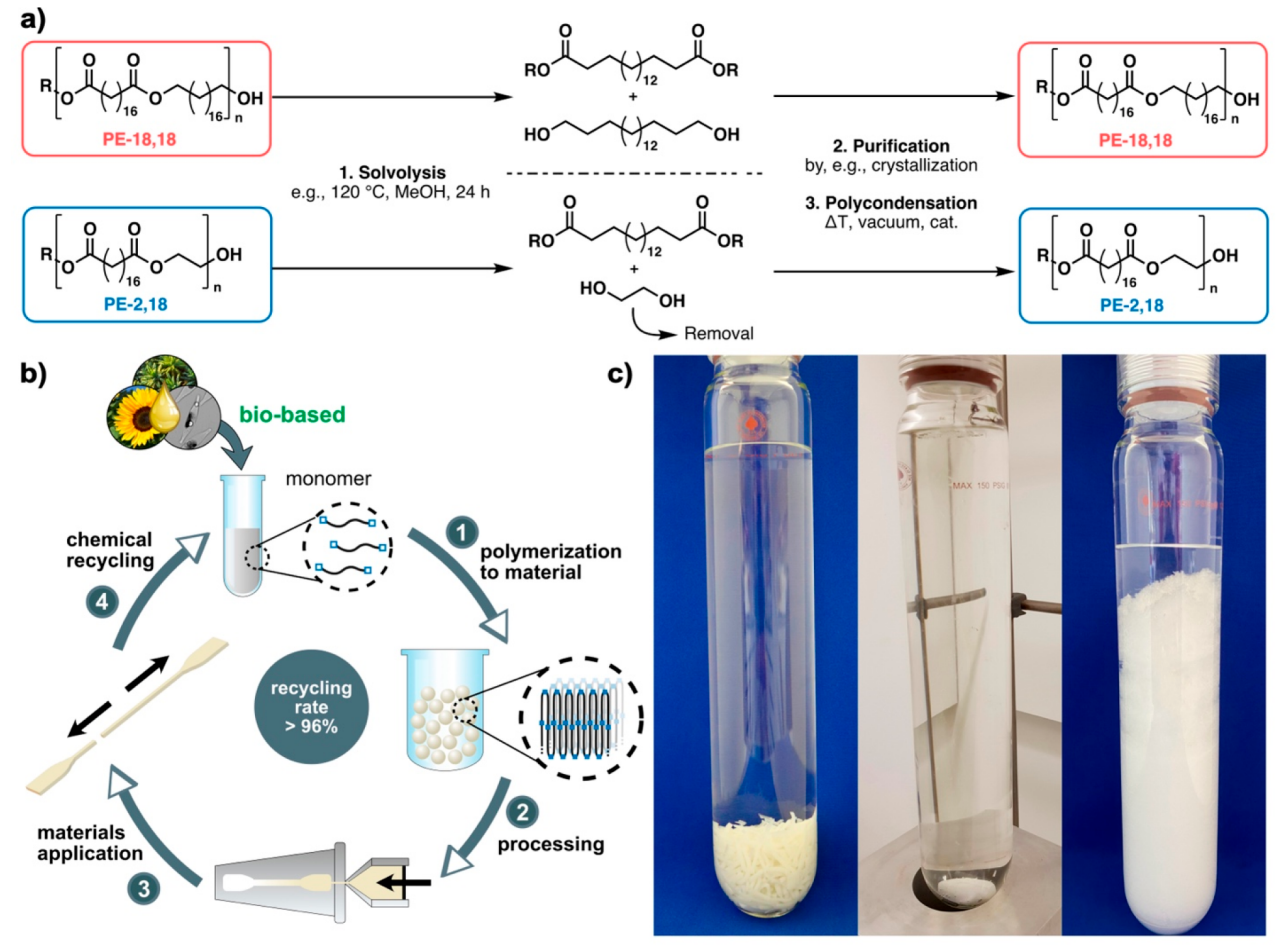
Closed-loop chemical recycling of polyethylene-like polyesters. (a) Schematic overview of the chemical recycling of PE-18,18 and PE-2,18 via solvolysis and repolymerization. (b) Closed-loop recycling concept for polyethylene-like polyesters, comprising (1) polymerization of renewable monomers to yield materials with polyethylene-like properties, (2) processing by compounding and injection molding, (3) materials application, and (4) chemical recycling by solvolysis at the end of service life. Adapted with permission from ref (3). Copyright 2021, Springer Nature. (c) Chemical recycling of PE-18,18 via methanolysis: glass pressure reactor filled with melt-processed PE-18,18, methanol, and a stir bar (left); homogeneous reaction mixture at 120 °C (center), and C_18_-monomer mixture crystallizing upon cooling of the reaction mixture at the end of the solvolysis process (right).

The solvolyzable in-chain functional groups of HDPE-like long-chain polyesters can act as predetermined breaking points and facilitate deconstruction of the polymer chain to recover the underlying long-chain monomers. This chemical recycling was demonstrated for PE-18,18 and also the long-chain polycarbonate PC-18.^3^ Alcoholysis under autogenous pressure (Figure 7a,c) proceeds within hours at temperatures of 120–180 °C and can optionally be accelerated by KOH as a depolymerization catalyst. Under such conditions, the polymer melt (*T*_m_ = 99 °C for PE-18,18) with progressing depolymerization gradually dissolves, yielding a homogeneous mixture. The long-chain monomers can be recovered from this reaction solution in quantitative yield by either the removal of solvent or crystallization. In fact, the long-chain aliphatic monomers’ pronounced ability to crystallize^3,55^ is a key to effective separation and recovery following solvolysis. Note that in the case of PE-18,18 methanolysis, a mixture of two long-chain monomers, namely, C_18_-dimethyl ester and C_18_-diol, is obtained. Due to their nonvolatility, the two building blocks cannot be separated easily, however, the mixture’s 1:1 stoichiometry renders it suitable for direct repolymerization. By comparison, ethanolysis of PC-18 yields crystallizable C_18_-diol and volatile diethylcarbonate, which can each be isolated. Polymers generated from the recovered monomers exhibit properties that are on par with the virgin starting polymers (Figure 7b). A similar solvolysis approach was also demonstrated for the aforementioned short–long-chain polyesters (e.g., PE-2,18) and PE-like polyesters containing multiple chain length dicarboxylic acid building blocks (e.g., PE-2,14 ± 2).

In view of the requirements of a real-life recycling scenario, the chemical recycling of PE-18,18 and PC-18 containing colorants from a model waste stream containing commercial polyolefins and PET was demonstrated.^3^ Selective depolymerization, leaving the other polymers, including PET intact, yielded very pure long-chain monomers.

## Degradability

At the end of use, a significant share of plastics leaks out of the collection system and becomes an environmental contaminant. While more effective waste management is clearly required, it is questionable that leakage can fully be eliminated.^52^ Therefore, nonpersistent behavior is a desirable property not only for polymers designed to rapidly biodegrade at the end of their service life in specific applications (e.g., agricultural films) but also for plastics in general as it can help to reduce the negative environmental impact of plastic leakage.

The biodegradation of a polymer material comprises two major steps: depolymerization to low-molar-mass building blocks, rate-determining for the overall process, and subsequent microbial mineralization of the depolymerization products to CO_2_ (or CH_4_ under anaerobic conditions), biomass, and water. Although the biodegradation of polyesters such as PBAT has been much studied, the biodegradation behavior of long-chain polyesters has been rather underexplored. Heise et al. subjected extruded PE-15 samples to a phosphate-buffered solution (pH 7.4) for a period of 2 years at 37 °C.^56^ Under these conditions, the crystalline (χ = 68%) and hydrophobic material proved to be remarkably stable (i.e., no mass loss, reduction of molar mass, or change in crystallinity was observed). Even the addition of a hydrolyzing enzyme did not lead to significant cleavage of the in-chain ester bonds. While this hydrolytic stability, which we also found for PE-18,18 in acidic and basic media,^46^ can be beneficial for certain applications, it at the same time impedes biodegradability, desirable as a backstop for plastic accumulation in the environment.

Abiotic hydrolytic degradation in the environment is expected to be slower than biodegradation under optimum conditions in general, but it can be less dependent on the specific microbial environment present. Therefore, it may compliment biodegradability as a means to prevent long-term persistency and accumulation of plastics pollutants. To this end, blending PE-18,18 with a small amount of hydrolytically labile long-chain poly(H-phosphonates)^57^ (PHP-18 or PHP-26, derived from the polycondensation of C_18_- or C_26_-diol and a dialkylphosphonate) was found to enable its hydrolytic degradability.^46^ Upon immersion of bulk injection-molded blend specimens in water for a few months, complete hydrolysis to the monomers of the PHP component occurred. Concomitantly, the PE-18,18 matrix is hydrolyzed to a significant extent throughout the bulk, likely catalyzed by the phosphoric acid liberated by the PHP’s hydrolysis. This leads to embrittlement and fragmentation of the specimens, which increases the surface area and, in combination with the observed molar mass reduction, is anticipated to accelerate and promote further degradation. As an alternative to this blending approach, acid functionalities can be incorporated into the polymer chain.^58^ Long-chain polyesters containing small amounts of sulfonic acid groups exhibit enhanced water uptake, which facilitates the acid-catalyzed cleavage of the in-chain ester groups. Note that ionic in-chain groups not only enable hydrolytic degradability but also benefit the surface properties of HDPE-like polymers allowing for printability on films.^58^

**Figure 8:**
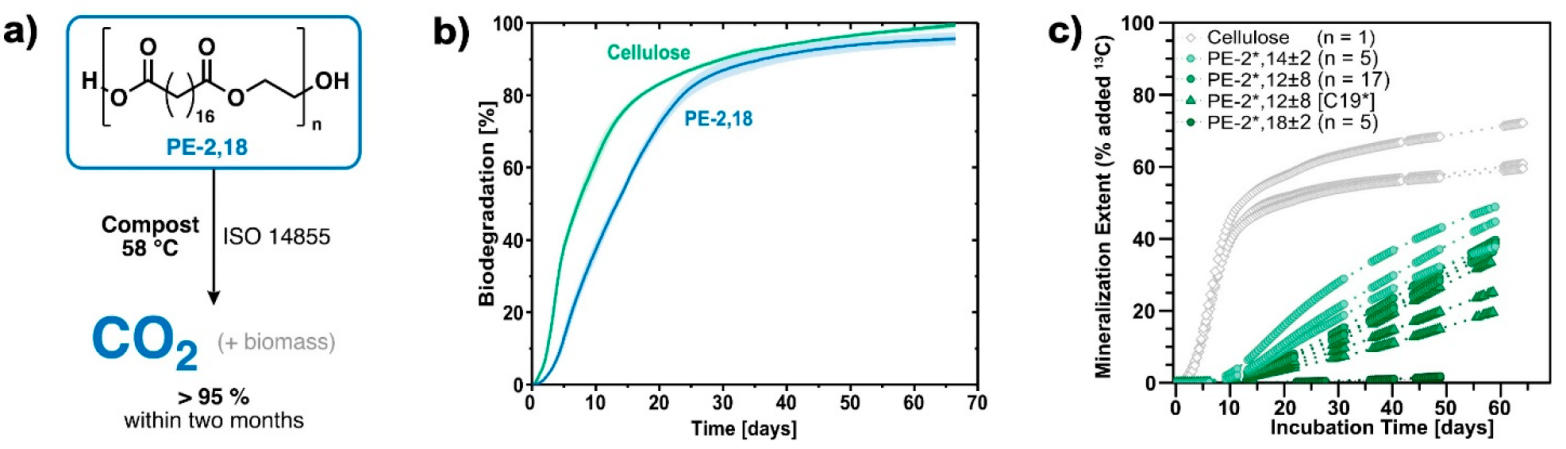
Biodegradation of PE-2.18. (a) Schematic of mineralization of PE-2,18 to CO_2_ in a respirometric test under industrial composting conditions according to standard ISO 14855. (b) Mineralization over time based on CO_2_ evolution measured under industrial composting conditions for PE-2,18 and for cellulose as a reference material. Shadows in light color correspond to standard deviations. Adapted with permission from ref (2). Copyright 2023, Wiley-VCH Verlag GmbH *&* Co. KGaA. (c) Soil biodegradation of ^13^C-labeled polyesters, studied for polyesters PE-2,X ± Y from mixtures of long-chain dicarboxylic acid monomers with different centers (X) and breadths of distributions (Y) of the monomer chain length. Adapted with permission from ref (1). Copyright 2023, Wiley-VCH Verlag GmbH *&* Co. KGaA.

Sander et al. in their development of high-throughput analysis methods on thin films of a series of aliphatic polyesters made from butanediol and C_4_- to C_18_-diacids (i.e., PE-4,4 to PE-4,18) found clear evidence for enzymatic hydrolysis even for the polyester containing the longest dicarboxylic acid, that is, PE-4,18.^59^ We found that the depolymerization of PE-2,18 to the monomer by a naturally occurring esterase in *in vitro* hydrolysis experiments proceeded completely to the monomers within days. Under the same conditions, PE-18,18 was much more stable, with monomer formation being barely detectable (<1%).^2^ Studies by BASF SE of the biodegradation under industrial composting conditions (58 °C, ISO standard 14855), as encountered in municipal composting facilities, revealed that despite its crystallinity the material under these conditions is biodegraded with mineralization above 95% within 2 months (Figure 8a,b). Thus, PE-2,18 fulfills standard EN 13432, which defines requirements that a packaging material has to meet in order to be certified as compostable (≥90% mineralization within 6 months).

The advantageous combination of HDPE-like material properties and the biodegradability of PE-2,18 is not limited to thermoplastics but can also be transferred to other classes of materials. Polyethylene waxes are employed on a large scale in multiple applications, and they are coming under increased scrutiny for their insufficient environmental degradability. We found that waxes based on the repeat unit motif of PE-2,18 (or 12,12) with an average *M*_n_ of 10^3^ to 10^4^ g mol^–1^ can have a profile of properties relevant for applications that compares well to their petrochemical PE-wax counterparts.^60^

For the aforementioned mixed-monomer chain-length polyesters, (PE-2,X ± Y)^1^ degradation was also studied in an agricultural soil. Compared to the harsh conditions in industrial compost in terms of a particularly broad microbial consortium being present and the elevated temperature (58 °C), this represents milder conditions of biodegradation. To accurately monitor the expected lower degradation rates and under these conditions unambiguously differentiate polymer carbon-derived CO_2_ from background CO_2_, isotope-selective quantification^61^ was employed. Polyesters fully ^13^C-labeled in the diol (C_2_) or in the dicarboxylate (C_19_) repeat unit were studied. Notably, polyesters with lower center dicarboxylic acids (e.g., PE-2,14 ± 2) or broader dicarboxylic acid distributions (e.g., PE-2,12 ± 8) exhibited significant mineralization within 2 months by the native soil microorganisms present. Even polyesters with relatively high center dicarboxylic acids (e.g., PE-2,18 ± 2) were slowly mineralized under these nearly natural conditions, indicating that long-chain polyesters have the potential to be nonpersistent when improperly released into natural environments (Figure 8c).

## Conclusions and Perspectives

While a long-chain polyester played a prominent role in Carothers’ seminal work that founded today’s polyester industry, for a long time afterward the interest in these polymers was restricted largely to the dependence of the melting point on monomer length. Studies of long-chain polyesters as materials were intensified only in this century, initially motivated primarily by their potentially biobased nature as an alternative to petrochemistry-based plastics such as polyethylene. More recently, the suitability of long-chain polyesters for chemical recycling and their biodegradability have moved into the focus.^2,3^ The advent of commercial sources of long-chain dicarboxylates in the past few years has also provided a new dynamic in the field. With their polyethylene-like solid-state structures, long-chain polyesters can possess sufficiently high crystallization temperatures for efficient melt processing and sufficiently high melting temperatures for the requirements of many applications. At the same time, the fact that their melting points are ≤140 °C as the theoretical limit (compared to *T*_m_ = 268 °C for polyethylene terephthalate) allows for energy-efficient processing. Also, solvolysis recycling can be performed under comparatively moderate conditions of ca. 120 to 180 °C, which may be further improved upon by enzymatic depolymerization processes. The propensity of long-chain compounds such as dicarboxylates for crystallization can enable recycling processes. Despite their crystalline and rather hydrophobic nature, long-chain polyesters can be biodegradable. Complete mineralization can occur within only 2 months under industrial composting conditions and also under less harsh conditions of home composting, and even in soil significant biodegradation has been observed.^1,2^ The rate of biodegradation varies strongly with the type of polyester repeat unit, in particular with the diol chain length. This offers opportunities for tuning degradation rates and stability toward degradation to the requirements of different applications and environments. In particular, stable materials which, however, unlike polyolefins, do not persist for decades or centuries if improperly released into the environment are achievable. The studies of long-chain polyesters reviewed here were performed on the laboratory or small pilot scale. Materials based on commercially available monomers, such as polyester-2.18,^2^ open the door for larger-scale developments and applications. Areas of interest comprise food packaging films and textile fibers,^62^ to name only two examples. Further studies among others should address, for a given long-chain polyester, upscaling and optimization with regard to the molecular weight of polymerization procedures, elaboration of melt rheologies and crystallization rates depending on polymer microstructure, the necessity of stabilization, and the optimization of processing parameters as well as key material properties for a given application. Connecting to this, on a more fundamental level an understanding of the relation of polymer microstructure and thermal histories to morphologies and (bio)degradation rates and mechanisms is of interest. Alternatively sourced monomers, generated from, for example, plastic or other waste, and the prospect of widely tunable biodegradation rates add to the versatility of prospects of this research arena.
